# Supplementary material for: Prevalence and Risk Factors of Eimeria spp. in Broiler Chickens from Pichincha and Santo Domingo de los Tsáchilas, Ecuador
Source: Pathogens. 2024 Jan 4;13(1):48. doi: 10.3390/pathogens13010048 (PMC10820216; doi:10.3390/pathogens13010048)
Supplement: Supplementary file 1 [file pathogens-13-00048-s001.zip › Table S1.pdf]

Table S1. General information and farm management

PARAMETER	CATEGORY	PARAMETER	CATEGORY
DEPENDENCE	Integrated	BED COMPOSTING METHOD	Fermentation
	Independent		None
COMPANY	Company 3	PRESENCE OF *Alphitubius diaperinus*	Yes
	Company 1		No
	Company 2	RAT CONTROL	Yes
	Company 4		No
	Company 5	INSECT CONTRO	Yes
	Others		No
FARM CAPACITY	Less than 26.000 birds	*Alphitubius diaperinus* CONTROL	Yes
	28.000 - 87.000 birds		No
	More than 88.000 birds	ANTICOCCIDIAL CONTROL METHOD	Vaccination
OTHER AGRICULTURAL ACTIVITIES	Yes		Anticoccidial in feed
	No		None
ALTITUDE	Below 1300mamsl	VACCINATION AGE	Eclotion
	Above 1300mamsl		First week
DENSITY (KG/M2)	Intermedium	VACCINE TYPE	Live vaccine
	Below		Attenuated vaccine
	High	VACCINATION CYCLES ON FARM	Less than 3 lots
DENSITY (BIRDS/M2)	Between 9.6 - 11		More than 4 lots
	Below than 9.5	LAST ACTIVE ANTICOCCIDIAL DRUG	
	More than 11.1	USED PROGRAM	Chemical – Ionophore
SHED TYPE	Controlled environment		Chemical
	Open air shed		Ionophore
ROOF MATERIAL	Galvalume		Ionophore – Chemical
	Asbestos	ROTATION TIME	Less than 6 months
	Zinc		More than 7 months
FLOOR MATERIAL	Cement	EFFECTIVE WITHDRAWAL TIME	Less than 5 days
	Soil		More than 6 days
ROOF SUPPORT MATERIAL	Metal	CLINICAL COCCIDIOSIS	Yes
	Wood		No
ADDITIONAL ILLUMINATION	No	WATER ANTICOCCIDIALS	Yes
	Yes		No
TYPE OF LIGHTING	Yellow	ACTIVE INGREDIENT	
	White	ADMINISTRATION AGE	
TYPE OF VENTILATION	Mechanic	TIME (days)	
	Natural	GENETIC	COBB
TYPE OF DRINKERS	Closed Systems		ROSS
	Open Systems	BIRG AGE	More than 46 days
ORIGIN OF WATER	River or canal		Less than 35 days
	Well		Between 36 to 45 days
	Drinking water	SEX	Male
SANITIZERS USED IN WATER	Chlorine		Female
	Chlorine and Hydrogen peroxide		Mixt
	Hydrogen peroxide	WEIGHT	Less than 2.5kg
	Three or more		More than 2.6kg
HYDROGEN PEROXIDE	Yes	FEED TYPE	Free (Ad libitum)
	No		Controlled
CHLORINE	Yes	REPOPULATION TIME	More than 68 days
	No		Less thana 60 days
TYPE OF FEEDERS	Mechanical		Between 61 to 67 days
	Manual	SANITARY EMPTYING	More than 18 days
FEED PRESENTATION	Flour		Between 8 to 15 days
	Pellet		Between 3 to 7 days
ORIGIN OF FEED	Manufacture	LIME	No
	Purchase		Yes
BEDDING MATERIAL	Rice husk	PHENOLIC	No
	Wood shavings		Yes
	Others	FORMALDEHYDE	No
BEDDING HUMIDITY	Less than 25%		Yes
	More than 26%	FLAMING	No
NUMBER OF BED USES	Two o three uses		Yes
	One use	QUATERNART AMMONIUM	No
	More than 4 uses		Yes
BED DEPTH	Between 10 to 15cm	OTHER DISINFECTANS	No
	Less than 10cm		Yes
	More than 15cm		
