# Supplementary material for: Prevalence and Risk Factors of Eimeria spp. in Broiler Chickens from Pichincha and Santo Domingo de los Tsáchilas, Ecuador
Source: Pathogens. 2024 Jan 4;13(1):48. doi: 10.3390/pathogens13010048 (PMC10820216; doi:10.3390/pathogens13010048)
Supplement: Supplementary file 1 [file pathogens-13-00048-s001.zip › Table S2.pdf]

Table S2. Relationship between oocyst per gram of feces (OPG) and *Eimeria* spp. species found with the characteristics and control measures applied in the studied farms.

	OPG			*E. acervulina*			*E. brunetti*			*E. maxima*			*E. necatrix*			*E. praecox*			*E. tenella*		
**Parameter**	**%**	**OPG media**	**P**	%	OR (95%CI)	P	%	OR (95%CI)	P	%	OR (95%CI)	P	%	OR (95%CI)	P	%	OR (95%CI)	P	%	OR (95%IC)	P
DEPENDENCE																					
Integrated	58%	5460	0.19	65.52	Reference		21.5	Reference		77	Reference		45.2	Reference		46.2	Reference		42.5	Reference	
Independent	42%	9052		77.27	2.13 (1.00-4.72)	0.039*	43.9	2.84 (1.35-6.08)	0.003**	85	2.16 (0.91-5.48)	0.083	63.6	2.21 (1.11-4.47)	0.016 *	68.2	2.48 (1.23-5.10)	0.009 **	68.2	3.22 (1.59-6.67)	0.001 **
FARM SIZE																					
Large	31%	4988.3		65.12	Reference		26.5	Reference		81	Reference		55.1	Reference		53.1	Reference		53.5	Reference	
Medium	35%	6804.6	0.38	71.43	7.86 (0.77-4.57)	0.15	25	0.92 (0.35-2.44)	1	79	1.46 (0.55-3.95)	0.252	50	0.82 (0.35-1.88)	0.7	57.1	1.18 (0.51-2.73)	0.698	42.9	0.85 (0.37-1.97)	0.698
Small	34%	8883.8		74.07	2.13 (0.86-5.37)	0.96	40.7	1.89 (0.77-4.82)	0.15	81	1.75 (0.63-4.99)	0.498	51.9	0.88 (0.38-2.05)	0.87	55.6	1.11 (0.47-2.58)	0.845	64.8	2.07 (0.88-4.96)	0.077
ALTITUDE																					
Below 1300MAMSL	77%	8338.1	0.000**	64.86	Reference		18.9	Reference		76	Reference		54.1	Reference		51.4	Reference		37.8	Reference	
Above 1300MAMSL	23%	2582.4		72.41	1.32 (0.56-3.02)	0.55	34.4	2.35 (0.91-6.85)	0.07	82	1.30 (0.50-3.22)	0.515	51.6	0.98 (0.44-2.16)	1	56.6	1.33 (0.60-2.94)	0.461	58.6	0.486 (0.21-1.09)	0.062
DENSITY (Kg/m$_2$)																					
Intermediate	29%	12113.61^{ab}		72.92	Reference		37	Reference		92	Reference		53.7	Reference			Reference		60.4	Reference	
Low	33%	14289.6^{a}	0.011*	84.31	2.44 (0.94-6.65)	0.05	33.3	0.83 (0.34-1.97)	0.2	86	1.22 (0.41-3.69)	0.81	56.9	1.08 (0.47-2.47)	0.85	62.8	1.27 (0.55-2.93)	0.565	60.8	0.68 (0.29-1.57)	0.567
High	38%	9579.94^{b}		55.56	0.82 (0.35-1.92)	0.69	22.2	0.54 (0.21-1.36)	0.69	65	0.55 (0.20-1.45)	0.19	46.3	0.86 (0.38-1.98)	0.85	48.2	0.87 (0.38-1.99)	0.846	40.7	1.26 (0.55-2.9)	0.339
DENSITY (Birds//m$_2$)																					
Between 9.6 and 11	34%	6128.24^{ab}		65.12	Reference		32.7	Reference		82	Reference	0.64	49	Reference		44.9	Reference		60.5	Reference	
Below 9.5	32%	10049.12^{a}	0.025*	80	2.97 (1.18-7.72)	0.012**	33.3	1.03 (0.43-2.50)	1	88	1.29 (0.45-3.67)		53.3	1.19 (0.52-2.71)	0.7	65	2.26 (0.98-5.31)	0.052	56.7	1.16 (0.51-2.64)	0.847
Above 11.1	34%	4847.69^{b}		64	1.33 (0.550-3.239)	0.54	26	0.73 (0.28-1.89)	0.51	72	0.75 (0.26-2.04)	0.645	54	1.22 (0.52-2.90)	0.69	54	1.44 (0.61-3.43)	0.424	44	0.68 (0.29-1.57)	0.424
TYPE OF POULTRY HOUSE																					
Controlled environment	17%	7812	0.63	66.67	Reference		22.2	Reference		67	Reference		51.9	Reference		59.3	Reference		40.7	Reference	
Open	83%	6774.9		71.43	1.07 (0.39-2.77)	1	32.6	1.69 (0.60-5.48)	1	83	1.94 (0.69-5.17)	0.21	52.3	1.02 (0.41-2.53)	1	54.6	0.83 (0.32-2.06)	1	56.4	1.69 (0.67-4.35)	0.391
ROOF MATERIAL																					
Galvalume	84%	7225.6		71.88	Reference		30.6	Reference		83	Reference		53.7	Reference		54.5	Reference		55.5	Reference	
Asbestos	9%	4494.6	0.2	57.14	0.61 (0.17-2.28)	0.38	28.6	0.91 (0.20-3.38)	1	64	0.48 (0.13-1.97)	0.31	50	0.86 (0.24-3.05)	1	57.1	1.11 (0.32-4.12)	1	42.9	0.67 (0.18-2.33)	0.578
Zinc	7%	6731.8		72.73	1.22(0.27-7.47)	1	36.4	1.29 (0.26-5.42)	0.74	73	0.71 (0.16-4.40)	0.7	36.4	0.49 (0.10-2.05)	0.35	63.6	1.46 (0.35-7.12)	0.755	45.5	0.74 (0.17-3.07)	0.757

FLOOR MATERIAL																					
Cement	49%	2614.1	0.000**	56.94	Reference		26.9	Reference		78	Reference		46.2	Reference		44.9	Reference		51.4	Reference	
Soil	51%	11127		82.71	4.28 (1.98-9.67)	0.000**	34.6	1.43 (0.69-3.00)	0.31	83	1.87 (0.83-4.36)	0.13	58	1.61 (0.82-3.17)	0.15	65.4	2.31 (1.17-4.64)	0.011 **	55.6	1.38 (0.71-2.71)	0.343
ROOF SOPPORT MATERIAL																					
Metal	39%	5701.6	0.28	57.63	Reference		25.8	Reference		75	Reference		50	Reference		41.9	Reference		54.2	Reference	
Wood	61%	7749.5		78.72	2.63 (1.26-5.56)	0.006**	34	1.48 (0.69-3.24)	0.3	84	1.79 (0.79-4.07)	0.17	53.6	1.15 (0.58-2.30)	0.75	63.9	2.44 (1.21-4.97)	0.009 **	53.2	1 (0.50-1.98)	1
ADDITIONAL ILLUMINATION																					
NO	29%	5888	0.21	72.5	Reference		32.6	Reference		88	Reference		52.2	Reference		56.5	Reference		67.5	Reference	
YES	71%	7383.7		69.91	1.36 (0.61-2.96)	0.46	30.1	0.89 (0.40-2.01)	0.81	78	1.11 (0.44-2.64)	0.34	52.2	1 (0.48-2.10)	1	54.9	0.56 (0.44-1.97)	0.863	48.7	0.67 (0.31-1.41)	0.295
VENTILATION TYPE																					
Mechanical	19%	7250.8	0.88	71.43	Reference		26.7	Reference		75	Reference		53.3	Reference		56.7	0.94 (0.38-2.27)		46.4	Reference	
Natural	81%	6881.2		70.4	1.07 (0.41-2.66)	1	31.8	1.28 (0.50-3.61)	0.67	82	1.61 (0.58-4.21)	0.33	51.9	0.95 (0.39-2.26)	1	55	Reference	1	55.2	1.50 (0.63-3.66)	0.418
TYPE OF DRINKERS																					
Closed systems	48%	7250	0.47	68.06	Reference		27.6	Reference		76	Reference		51.3	Reference		54	Reference		47.2	Reference	
Open systems	52%	6677.2		72.84	1.35 (0.66-2.79)	0.4	33.7	1.33 (0.64-2.79)	0.49	84	1.72 (0.77-3.97)	0.19	53	1.07 (0.55-2.09)	0.88	56.6	1.11 (0.57-2.19)	0.752	59.3	1.69 (0.86-3.33)	0.114
WATER SOURCE																					
River or canal	25%	3506.05[ab]	0.045*	51.28	Reference		38.5	Reference		64	Reference		46.2	Reference		38.5	Reference		53.9	Reference	
Well	67%	8734.63[a]		79.21	2.79 (1.215-6.46)	0.009**	27.1	0.60 (0.26-1.40)	0.22	87	2.57 (1.04-6.33)	0.026 *	55.1	1.43 (0.64-3.21)	0.36	63.6	2.77 (1.23-6.41)	0.008 **	53.5	0.87 (0.39-1.94)	0.852
Potable water	8%	2603.85[b]		61.54	1.51 (0.36-6.97)	0.75	38.5	1 (0.21-4.27)	1	77	1.85 (0.38-12.17)	0.51	46.2	1 (0.23-4.22)	1	38.5	1 (0.21-4.27)	1	53.9	1 (0.24-4.33)	1
SANITIZERS USED IN WATER																					
Chlorine	11%	3126.5	0.48	64.71	Reference		17.7	Reference		71	Reference		52.9	Reference		52.9	Reference		35.3	Reference	0.14
Chlorine and peroxide	69%	7679.4		67.27	1.12 (0.31-3.63)	1	35.5	2.55 (0.65-14.66)	0.18	80	1.49(0.37-5.12)	0.54	50.9	0.92 (0.29-2.92)	1	54.6	1.07 (0.33-3.37)	1	56.4	2.35 (0.73-8.33)	0.123
Peroxide	2%	4050		66.67	1.09 (0.05-74.91)	1	33.3	2.22 (0.03-58.14)	0.51	67	0.84 (0.04-58.86)	1	100	0.28 (infinite)	0.24	66.7	1.73 (0.08-117.70)	1	33.3	0.92 (0.01-21.37)	1
More than 3 products	18%	6910.2		91.3	1.42 (0.32-6.15)	0.74	20.7	1.21 (0.22-8.70)	1	91	1.58 (0.31-7.76)	0.72	51.7	0.95 (0.24-3.71)	1	58.6	1.25 (0.32-4.94)	0.765	56.5	1.48 (0.37-6.29)	0.555
PEROXIDE																					
YES	71%	7679.4	0.22	65.49	Reference		34.5	Reference	0.07	76	Reference		49.6	Reference	0.73	53.1	Reference	0.863	54.9	Reference	
NO	29%	5315		85	0.91 (0.41-1.97)	0.86	21.7	2.13 (0.92-5.33)		93	1.16 (0.48-2.72)	0.69	58.7	0.85 (0.41-1.75)		60.9	0.90 (0.43-1.87)		50	1.87 (0.90-3.94)	0.086
CHRORINE																					
YES	80%	7069.9	1	66.93	Reference	0.68	33.1	Reference	0.29	77	Reference		51.2	Reference	0.69	54.3	Reference	0.693	53.5	Reference	0.332
NO	20%	6478.9		88.46	0.79 (0.29-1.97)		21.9	1.76 (0.67-5.22)		96	0.95 (0.31-2.56)	1	56.3	0.82 (0.34-1.91)		59.4	0.82 (0.34-1.91)		53.9	1.48 (0.63-3.51)	
TYPE OF FEEDERS																					
Mechanical	55%	6942.5	0.32	69.14	Reference		24.1	Reference		80	Reference		54	Reference		57.5	Reference		46.9	Reference	

Manual	45 %	6961.2		72.22	1.44 (0.70-3.01)	0.31	38.9	1.99 (0.96-4.20)	0.06	81	1.40 (0.62-3.25)	0.45	50	0.85 (0.43-1.67)	0.64	52.8	0.83 (0.42-1.63)	0.631	61.1	2.02 (1.02-4.02)	0.038*
FEED PRESENTATION																						
Flour	25 %	2908.8	0.05	64.71	Reference		32.5	Reference		85	Reference		55	Reference		50	Reference		64.7	Reference	
Pelletized	75 %	8309.7		72.27	2.12 (0.94-4.76)	0.05	30.3	0.90 (0.39-2.13)	0.84	79	1.42 (0.56-3.45)	0.39	51.3	0.86 (0.39-1.88)	0.72	57.1	1 (0.61-2.91)	0.466	50.4	0.83 (0.38-1.81)	0.715
FEED ORIGIN																						
Manufactured	76 %	4939.5	0.002**	62.61	Reference		22.3	Reference		76	Reference		47.9	Reference		49.6	Reference		43.5	Reference	
Purchased	24 %	13356		94.74	12.11 (2.89-108.61)	0.000**	57.9	4.73 (2.06-11.16)	0.000**	95	6.94 (1.64-63.08)	0.003 **	65.8	2.08 (0.92-4.87)	0.064	73.7	2.83 (1.21-7.12)	0.009 **	84.2	7.48 (2.81-23.55)	0.000**
NUMBER OF BEDDING USAGE																						
Two or three uses	33 %	7642.6		66.04	Reference		41.5	Reference		77	Reference		47.2	Reference		56.6	Reference		62.3	Reference	
One use	52 %	6231.1	0.46	76.32	1.24 (0.55-2.77)	0.57	25.6	0.49 (0.22-1.09)	0.06	87	1.21 (0.47-3.03)	0.67	53.7	1.29 (0.62-2.75)	0.49	54.9	0.93 (0.44-1.98)	0.861	51.3	0.55 (0.25-1.18)	0.113
More than 4 uses	15 %	7883.3		62.5	0.86 (0.28-2.69)	0.8	25	0.47 (0.13-1.51)	0.21	67	1.70 (0.50-5.56)	0.4	58.3	1.56 (0.53-4.70)	0.46	54.2	0.91 (0.31-2.69)	1	41.7	0.44 (0.14-1.29)	0.137
BEDDING DEPTH																						
From 10 to 15cm	9 %	1465.3		60	Reference		33.3	Reference		73	Reference		26.7	Reference		66.7	Reference		46.7	Reference	
Less than 10cm	40 %	7230.9	0.35	68.75	1.46 (0.37-5.35)	0.55	32.8	0.98 (0.26-4.12)	1	81	1.57 (0.31-6.58)	0.49	62.5	4.93 (1.17-21.56)	0.019 *	60.9	0.78 (0.19-2.88)	0.774	59.4	1.66 (0.46-6.11)	0.399
More than 15cm	50 %	7755.6		74.32	1.46 (0.38-5.19)	0.56	28.8	0.81 (0.22-3.36)	0.76	81	1.09 80.23-4.24)	1	48.8	2.59 (0.69-12.11)	0.16	48.8	0.48 (0.12-1.71)	0.264	50	0.98 (0.28-3.52)	1
BEDING COMPOSTING METHOD																						
Fermentation	53 %	8010.2	0.19	70.51	Reference		32.1	Reference		79	Reference		48.8	Reference		56	Reference		56.4	Reference	
None	47 %	5764.7		70.67	1.27 (0.62-2.63)	0.5	29.3	0.88 (0.42-1.82)	0.73	81	1.54 (0.68-3.58)	0.34	56	1.33 (0.68-2.62)	0.43	54.7	0.95 (0.48-1.86)	0.875	50.7	0.93 (0.48-1.82)	0.824
PRESENCE OF *Alphitubius diaperinus*																						
No	40 %	8734.2	0.058.	58.73	Reference		27	Reference		68	Reference		47.6	Reference		46	Reference		41.3	Reference	
Yes	60 %	4233.7		78.89	1.99 (0.96-4.15)	0.06	33.3	1.35 (0.64-2.92)	0.48	89	2.31 (1.02-5.32)	0.033 *	55.2	1.35 (0.68-2.69)	0.42	61.5	1.86 (0.93-3.74)	0.073	62.2	1.98 (1.00-4.00)	0.051*
WATER MEDICATED WITH ANTICOCCIDIALS																						
No	89 %	6248	0.06	67.88	Reference		27	Reference		78	Reference		50.4	Reference		54.6	Reference		49.6	Reference	
Yes	11 %	12450		93.75	2.57 (0.68-14.51)	0.18	61.1	4.21 (1.38-13.83)	0.006**	100	2.53 (0.55-23.80)	0.37	66.7	1.96 (0.64-6.75)	0.22	61.1	1.30 (0.43-4.21)	0.802	87.5	3.73 (1.10-16.32)	0.023*
AGE																						
More than 46 days	33 %	3021[b]	0.006**	56	Reference		26.9	Reference		70	Reference		36.5	Reference		40.4	Reference		44	Reference	
Less than 35 days	34 %	12388[a]		92.31	6.73 (2.32-22.64)	0.001**	31.5	1.24 (0.50-3.16)	0.67	94	4.69 (1.48-17.84)	0.004 **	57.4	2.32 (1.00-5.51)	0.011 **	72.2	3.79 (1.58-9.41)	0.002 **	55.8	1.57 (0.69-3.66)	0.525
Between 36 and 45 days	33 %	5266[b]		62.75	1.30 (0.56-3.04)	0.56	34	1.39 (0.56-3.52)	0.53	76	1.35 (0.54-3.44)	0.53	62.3	2.84 (1.21-6.83)	0.035 *	52.8	1.65 (0.71-3.85)	0.242	60.8	1.91 (0.83-4.49)	0.12
FEEDING TYPE																						
Free (Ad libitum)	36 %	9226.3	0.26	106.9	Reference		25.9	Reference		81	Reference		55.2	Reference		65.5	Reference		43.1	Reference	

Controlled	64%	5644.4		48.42	0.42 (0.18-0.93)	0.022*	33.7	1.45 (0.67-3.23)	0.37	80	0.71 (0.29-1.67)	0.44	50.5	0.83 (0.41-1.66)	0.62	49.5	0.52 (0.25-1.06)	0.068	60	1.70 (0.85-3.46)	0.138
RESTOCKING TIME																					
More than 68 days	23%	7170.1	0.25	86.67	Reference		30.6	Reference		97	Reference		58.3	Reference		61.1	Reference		63.3	Reference	
Less than 60 days	40%	10175		58.33	0.54 (0.20-1.42)	0.2	35	1.22 (0.47-3.32)	0.82	72	0.61 (0.19-1.80)	0.47	43.3	0.55 (0.22-1.36)	0.2	41.7	0.46 (0.18-1.14)	0.091	55	1.09 (0.44-2.71)	0.629
Between 61 to 67 days	38%	3434.3		74.6	1.13 (0.40-3.11)	0.81	27	0.84 (0.31-2.32)	0.82	81	1.03 (0.31-3.21)	1	57.1	0.95 (0.38-2.36)	1	65.1	1.18 (0.46-2.99)	0.828	47.6	0.82 (0.33-2.00)	0.836
SANITARY EMPTY PERIOD																					
More than 18 days	17%	9256.7	0.18	88.89	Reference		48.2	Reference		89	Reference		63	Reference		66.7	Reference		66.7	Reference	
8 to 15 days	50%	6064.6		59.49	0.19 (0.03-0.69)	0.005**	32.9	0.53 (0.20-1.42)	0.17	72	0.36 (0.06-1.25)	0.11	46.8	0.52 (0.19-1.38)	0.18	51.9	0.54 (0.19-1.46)	0.262	54.4	0.60 (0.21-1.61)	0.367
3 to 7 days	33%	7097.6		78.72	0.29 (0.05-1.19)	0.09	18.9	0.26 (0.08-0.78)	0.009 **	89	0.48 (0.08-2.07)	0.36	54.7	0.71 (0.24-2.02)	0.63	54.7	0.61 (0.20-1.74)	0.345	44.7	0.33 (0.11-0.95)	0.033 *
QUICKLIME																					
No	27%	5307	0.2	48.84	Reference		37.2	Reference		65	Reference		46.5	Reference		51.2	Reference		58.1	Reference	
Yes	73%	7560.4		79.09	3.12 (1.41-6.94)	0.002**	28.5	0.67 (0.30-1.52)	0.34	86	2.41 (1.01-5.67)	0.033 *	54.3	1.36 (0.64-2.94)	0.48	56.9	1.26 (0.59-2.70)	0.591	51.8	0.70 (0.32-1.49)	0.373
PHENOLICS																					
No	60%	5024.3	0.22	65.17	Reference		36.8	Reference		80	Reference		48.4	Reference		48.4	Reference		65.2	Reference	
Yes	20%	9581.3		78.13	2.26 (0.84-6.84)	0.09	34.4	0.90 (0.35-2.23)	0.84	84	1.82 (0.60-6.72)	0.33	53.1	1.21 (0.50-2.92)	0.69	65.6	2.02 (0.82-5.19)	0.105	50	0.64 (0.26-1.55)	0.305
FORMALDEHYDE																					
No	55%	6751.4	0.44	70.37	Reference		36.8	Reference		84	Reference		48.3	Reference		52.9	Reference		61.7	Reference	
Yes	25%	4913.3		65	0.98 (0.42-2.34)	1	35	0.93 (0.39-2.16)	1	75	0.84 (0.32-2.28)	0.82	52.5	1.18 (0.52-2.69)	0.71	52.5	0.99 (0.44-2.24)	1	60	1.11 (0.49-2.57)	0.848
FLAMED																					
No	59%	4035	0.011*	61.36	Reference		31.9	Reference		78	Reference		44.7	Reference		45.7	Reference		54.6	Reference	
Yes	41%	11164		83.08	3.61 (1.61-8.65)	0.001**	29.2	0.88 (0.41-1.85)	0.86	83	1.77 (0.76-4.36)	0.18	63.1	2.11 (1.06-4.26)	0.025 *	69.2	2.65 (1.31-5.51)	0.004 **	52.3	1.05 (0.53-2.08)	1
QUATERNARY AMMONIUM																					
No	89%	6961.7	0.78	71.85	Reference		34.8	Reference		82	Reference		53.2	Reference		55.3	Reference		51.9	Reference	
Yes	11%	6866		61.11	0.71 (0.23-2.33)	0.59	50	2.51 (0.82-7.72)	0.1	67	0.54 (0.17-1.91)	0.25	44.4	0.71 (0.23-2.12)	0.62	55.6	1.01 (0.34-3.13)	1	66.7	2.02 (0.66-6.94)	0.215
OTHER DISINFECTANTS																					
No	94%	4008.3	0.19	70.83	Reference		32	Reference		80	Reference		52.7	Reference		52.7	Reference		54.9	Reference	
Yes	6%	7127.3		66.67	0.94 (0.19-6.06)	1	11.1	0.27 (0.01-2.09)	0.28	89	2.42 (0.31-111.03)	0.69	44.4	0.72 (0.14-3.49)	0.74	44.4	0.72 80.14-3.49)	0.738	33.3	0.45 (0.07-2.21)	0.317

** Differences in farm characteristics and/or control measures with $p \leq 0.01$

* Differences in farm characteristics and/or control measures with $p \leq 0.05$

CI: Confidence Interval. Different letters indicate significant differences.
